# Variation in Number and Formation of Repeat Sequences in the rDNA ITS2 Region of Five Sibling Species in the *Anopheles barbirostris* Complex in Thailand

**DOI:** 10.1673/031.011.13701

**Published:** 2011-10-17

**Authors:** Yasushi Otsuka

**Affiliations:** Department of Infectious Disease Control, Faculty of Medicine, Oita University, Hasama, Yufu, Oita 879-5593, Japan

**Keywords:** concerted evolution, repetitive sequences, sibling species members

## Abstract

Repeat sequences of approximately 100 base pairs in length were found in the rDNA ITS2 region of *Anopheles barbirostris* van der Wulp (Diptera: Culicidae) species A1, A2, A3, A4, and *An. campestris*-like in the *An. barbirostris* complex. Variation in the number of repeats was observed among the five sibling species. Specifically, 10 repeats were observed in A1, eight in A2, A4, and *campestris*-like, and three in A3. Based on similarities in the sequences of the repeats, related repeats were classified into nine groups. Although A2, A4, and the *campestris*-like species had the same number of repeats, the ITS2 region of the three species contained different groups of repeats. Excluding the repeat sequences facilitated good alignment of the ITS2 region in the five sibling species. Phylogenetic analyses of the 95 isolines were compared with results obtained from mitochondrial genes (COI and COII). The results revealed marked differences among the five sibling species, particularly regarding the ITS2 region of A3, which was more distinct from the other four species than COI and COIL Repeat sequences in the ITS2 region of other *Anopheles* species retrieved from GenBank also were analyzed. New repeat sequences were found in *An. beklemishevi* Stegnii and Kabanova, *An. crucians* Wiedemann and *An. funestus* Giles, suggesting that the occurrence of repeat sequences in the ITS2 region are not rare in anopheline mosquitoes.

## Introduction

*Anopheles* (*Anopheles*) *barbirostris* van der Wulp and *An. campestris* Reid (Diptera: Culicidae) both belong to the Barbirostris subgroup of the Myzorhynchus series, and are natural vectors of both malaria, due to *Plasmodium vivax* Grassi and Feletti (Haemosporida: Plasmodidae), and filariasis, caused by periodic *Brugia malayi* Brug (Spirurida: Onchocercidae) in Malaysia and Indonesia (Reid 1968; Atomosoedjono et al. 1976; Kirnowardoyo 1985). In addition, these mosquitoes are also suspected vectors of malaria and/or filariasis in Thailand (Iyengar 1953; Griffith 1955), where they may be natural vectors of *P. vivax* in the Aranyaprathet district of Sa Kaeo province (Limrat et al. 2001; Apiwathnasorn et al. 2002). Sattabongkot et al. (2004) also considered that these vectors play an important role in increasing *P. vivax* infections in Thailand. In addition, the overlapped adult morphology between *An. barbirostris* and *An. campestris* has led to problems in species identification, particularly when using damaged scales of wild-caught females in the field (Harrison and Scanlon 1975). Recently, at least five species members of the *An. barbirostris* complex, namely *An. barbirostris* species A1, A2, A3, A4, and *An. campestris*-like, have been discovered in Thailand using cytogenetic and molecular markers and crossing experiments (Saeung et al. 2007, 2008; Suwannamit et al. 2009; Thongsahuan et al. 2009).

The high interspecific divergence and low degree of intraspecific divergence associated with the internal transcribed spacer 2 (ITS2) region of ribosomal DNA (rDNA) has been applied widely to distinguish between closely related *Anopheles* species, e.g., Maculipennis group (Porter and Collins 1991; Cornel et al. 1996; Proft et al. 1999) and the *An. culicifacies* complex (Goswami et al. 2005). In previous molecular analyses of the five species in the *An. barbirostris* complex, ITS2 assays were capable of distinguishing between closely related species (Saeung et al. 2007, 2008; Suwannamit et al. 2009; Thongsahuan et al. 2009). However, unlike most *Anopheles* species, in which the ITS2 region ranges from 200 to 600 base pairs (bp) (Wilkerson et al. 2004), the ITS2 region of the five species of the *An. barbirostris* complex is not only longer than 900 bp, but it also varies between these species. The primary objective of this study was to characterize the extra length and size variation of ITS2 in the *An. barbirostris* complex resulting from repeat sequences. Existence of the repeat sequences made it difficult to align the ITS2 sequences of the five species. However, by excluding the repeat sequences, clear alignment of the ITS2 region could be obtained. Based on these sequence alignments, phylogenetic analyses of the five species were conducted and compared with phylogenies that were estimated using the mitochondrial cytochrome *c* oxidase subunits I and II (COI and COII). *Anopheles fluminensis* also included three repeats (125 bp each) in the ITS2 region (Brelsfoard et al. 2006), suggesting that other *Anopheles* species may also have repeat sequences in this region. Therefore, another objective of this study was to interpret the present findings within the context of repeat sequences in the ITS2 region of other anopheline mosquitoes deposited in GenBank.

## Materials and Methods

The ITS2, COI, and COII sequences of 95 isolines of *An. barbirostris* species A1 (n = 39), A2 (n = 15), A3 (n = 3), A4 (n = 3), and *An. campestris*-like (n = 35) were obtained from the GenBank database (Supplementary Table). The repeat sequences of the ITS2 region were aligned using the MARNA web server, based on the primary sequence and secondary structure as a default setting (Siebert and Backofen 2005; www.bioinf.unifreiburg.de/Software/MARNA/index.html). Phylogenetic trees were estimated from the repeat sequence data using the neighbor joining (NJ) and bootstrapping algorithms implemented in the MEGA software package version 4 (Tamura et al. 2007). Genetic distances were estimated by the Jukes-Cantor model with pairwise deletion of gaps.

Phylogenetic trees of all 95 isolines of the *An. barbirostris* complex were generated based on ITS2, COI, and COII sequences using distance, maximum likelihood (ML), and maximum parsimony (MP) methods. To analyze the ITS2 region, the repeat sequences were removed and the remaining sequences were aligned by Clustal W (Thompson et al. 1994). Combined COI and COII sequences were aligned using the sequences of *An. gambiae* Giles (NC_002084) and *An. pullus* Yamada (AY444349 and AY444350) as outgroups. The phylogenetic trees of ITS2 were left unrooted, because an outgroup with an easily aligned ITS2 region was not available and gaps were excluded from the alignments. For the distance analyses, the NJ algorithm was used, as described above, except for gap treatment. For ML, the best-fit models for the ITS2 and COI+COII sequences were selected using the hierarchical likelihood ratio test implemented in Modeltest 3.06 (Posada and Crandall 1998). The models selected were HKY+Γ for ITS2 and GTR+I+Γ for COI+COII. The ML trees for the selected models were generated by PhyML 3.0 (Guindon and Gascuel 2003). For the MP analysis, the trees were generated using the default heuristic search option in PAUP* 4.0 b10, with ten random-addition sequence replicates (Swofford 1998). Bootstrap analyses for the 1000 replicate datasets were performed for each tree to assess the statistical support for the nodes. Dot plot analyses were conducted to scan the ITS2 sequences of *Anopheles* species in GenBank for repeat sequences using the BioEdit program (Hall 1999). However, since short repeats were difficult to identify on plots, only repeat sequences longer than 30 bp were detected.

**Figure 1.**
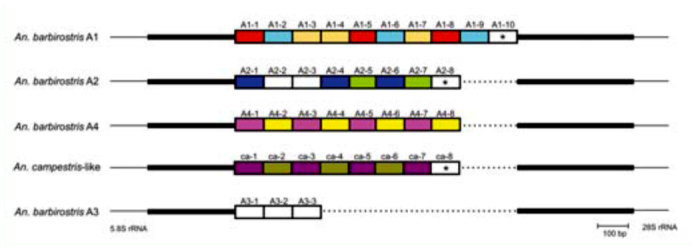
ITS2 characteristics of five sibling species in the *Anopheles barbirostris* complex. Each repeat unit is represented by a rectangle, and the designated name of each repeat is shown above the rectangle. Colors of the repeats correspond with the groups shown in Figure 3; white-colored repeats did not cluster with any of these groups. Despite belonging to different species, the repeats shown with an asterisk (A1-10, A2-8, and ca-8) clustered with high associated bootstrap values. The ITS2 region, except for the repeat sequences, is shown by the bold line. The 5.8 and 28S rRNA genes flanking the ITS2 region are shown by a thin line. Dots indicate gaps. High quality figures are available online.

## Results

### Repeat sequences in the ITS2 region of the five species in the *An. barbirostris* complex

Comparisons of ITS2 sequence alignments revealed that all five sibling species in the *An. barbirostris* complex contained sequences repeated tandemly, with each repeat sequence ∼ 100 bp long. *An. barbirostris* species A1 had ten repeats; A2, A4, and *An. campestris*-like had eight; and A3 had three (Figure 1). The sequences flanking the repeats in the five species were conserved, suggesting that the repeats were located at the same position. Since substitutions and indels had occurred among the repeat sequences, the relationships among the repeats were examined. First, the ITS2 sequences representing each of the five species (A1, AB331555; A2, AB331551; A3, AB362232; A4, AB373939; *An. campestris*-like, AB331563) were selected according to frequency of occurrence in each species. In this way, the 37 repeat sequences extracted from the ITS2 sequences of the five species were named; A1-1 to A1-10 (10 repeats), A2-1 to A2-8 (8 repeats), A3-1 to A3-3 (3 repeats), A4-1 to A4-8 (8 repeats) in the *An. barbirostris* species, and ca-1 to ca-8 (8 repeats) in the *campestris*-like species (Figure 1). The repeats were composed of inverted sequences, suggesting that they either formed secondary structures by themselves or in conjunction with other repeats. Most of the repeats had conserved sequences at both ends; GGGTG at the 5.8S rRNA-end and CA(C/T)CC at the 28S rRNA-end.

**Figure 2.**
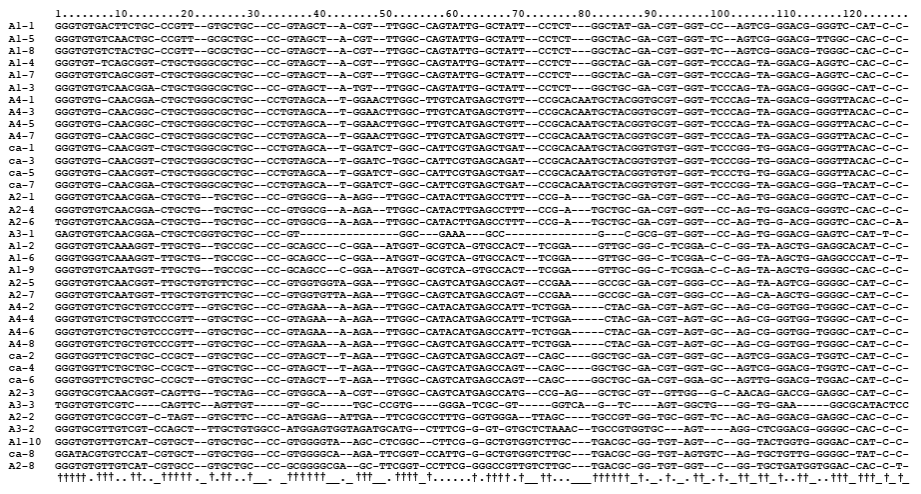
Alignment of the 37 repeat sequences from the ITS2 region of each of the five sibling species in the *Anopheles barbirostris* complex using the MARNA web server. Dashes indicate indels. Corresponding † indicate positions at which nucleotides were conserved in more than 50% of the repeats. High quality figures are available online.

**Figure 3.**
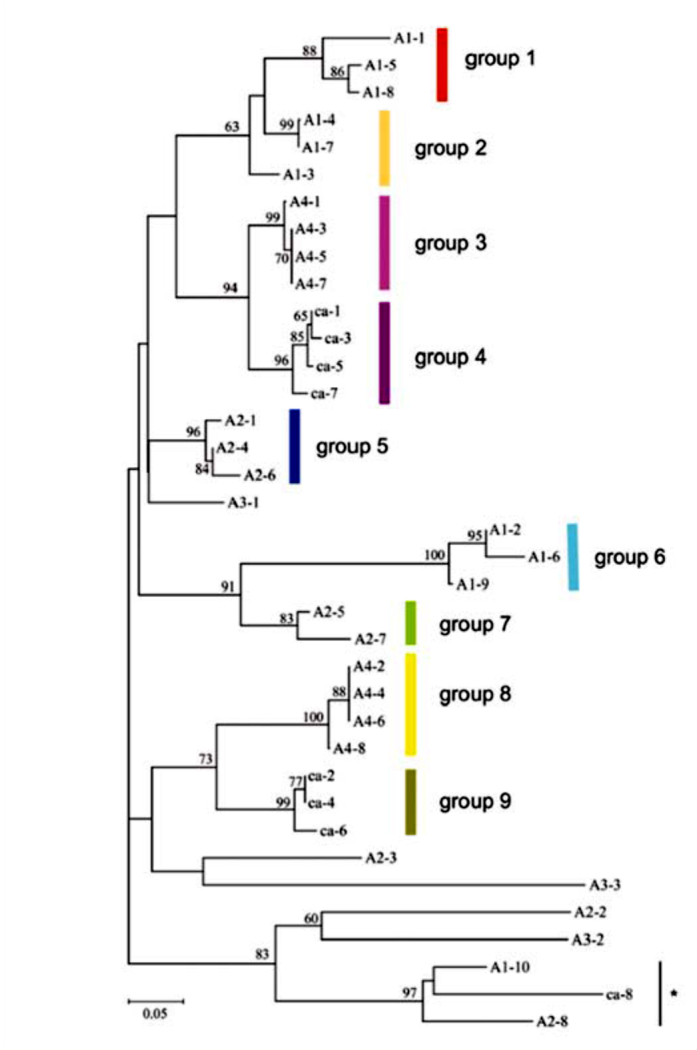
Neighbor-joining tree, constructed using data from the 37 repeat sequences of the ITS2 region in each of the five sibling species in the *Anopheles barbirostris* complex. Numbers above branches indicate the bootstrap value (> 50%). Groups were formed based on pairwise genetic distances of less than 0.1 between repeats, with some exceptions. Although the distance between A1-1 and A1–5 was 0.109, A1-1 was grouped with A1–5 and A1–8 because this inclusion was supported by a high bootstrap value. The distances between the three repeats (ca-1, ca-5, and ca-7) in group 4 and those in group 3 were less than 0.1. However, these two groups were grouped separately based on high bootstrap values. Groups are indicated by color bars to the right of the tree. The colors correspond to those used for the groups of repeats in Figure 1. High quality figures are available online.

Alignments based on primary sequence and secondary structure were performed (Figure 2), and an NJ tree was constructed based on the alignments (Figure 3). In order to examine the structural characteristics of the repeats in each species, sequences separated by genetic distances of less than 0.1 were classified as the same group. Nine groups were formed, and seven repeats remained ungrouped (Figure 3). The groups of repeats were correlated with species. The three ungrouped repeats, A1-10, A2-8, and ca-8, however, were clustered in the NJ tree with a high bootstrap value. These repeats were located closest to the 28S rRNA. Species A4 and *campestris*-like had eight similar repeat sequences consisting of tandem units that comprised one repeat from groups 3 and 8, and one from groups 4 and 9, respectively. The NJ tree demonstrated that group 3 and 8 were the most closely related to groups 4 and 9, respectively. However, the *campestris*-like species had ca-8, which was not found among any of the groups in the A4 and *campestris*-like species. A2 also included eight repeats, which had a different structure from those of the A4 and *campestris*-like species. A1 had 10 repeats belonging to groups 1, 2, and 6. A3 had three repeats, two of which were short in length; A3-1 and A3-3 were only 74 bp and 81 bp long, respectively, while the other 35 repeats were 95–112 bp in length. The three repeats of A3 had diverged considerably from one another, and did not belong to any group. In the NJ tree, A3-1 was placed closely to group 5, which included A2-1. In addition, A3-2 and A3-3 were grouped closest to A2-2 and A2-3, respectively, indicating that the array of the three repeats, A3-1, A3-2, and A3-3 was distantly related to A2-1, A2-2, and A2-3.

### Phylogenetic relationship between the five sibling species of the *An. barbirostris* complex

**Figure 4.**
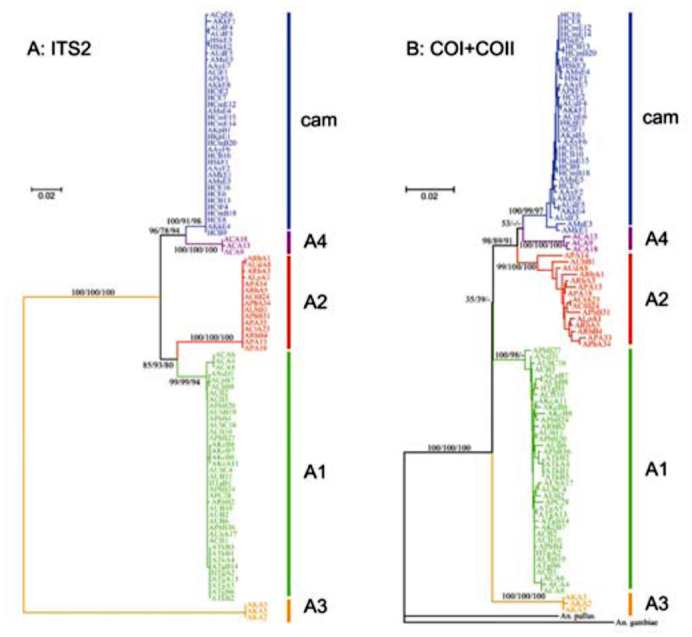
NJ trees of the 95 isolines of the five sibling species in the *Anopheles barbirostris* complex based on analysis of (A) the ITS2 region, with the repeat sequences excluded, and (B) combined sequences of COI and COII. Values at tree nodes indicate the level of support estimated by bootstrap replicates for distance, ML and MP methods, respectively, with dashes indicating a lack of support for the analysis. Colors indicate the isolines obtained in each species. High quality figures are available online.

Phylogenetic trees of the 95 isolines were generated based on the alignment of ITS2 sequences (Figure 4A). Each species was clustered in all of the trees and very little intraspecific variation was observed. A3 was placed separately from the other four species. Phylogenetic trees also were constructed based on the combined sequences of the mitochondrial COI and COII genes (Figure 4B). With the exception of the A1 isoline (APbB27) in the ML analysis, all species were separated. However, the relationships between A2, A4, and *campestris*-like were not clearly estimated. Marked differences between the trees of the two regions were observed. First, A3 was placed more closely to the other four species in the trees of the mitochondrial genes than in those of ITS2. Second, A2 was related closely to A1 in ITS2, but clustered with A4 and *campestris*-like in the mitochondrial genes.

### Repeats in the ITS2 region of other *Anopheles* species

**Figure 5.**
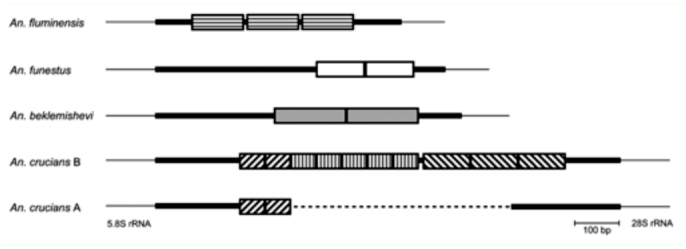
Schematic diagram of the ITS2 region in *Anopheles fluminensis* (DQ328638), *Anopheles funestus* (AF062512), *Anopheles beklemishevi* (AY593958), and *Anopheles crucians* B (AY386963) and A (AY245553). Repeats are shown by rectangles, with different shading patterns indicating no sequence similarity. The ITS2 region, excluding the repeat sequences, is shown by a bold line. The 5.8 and 28S rRNA genes flanking the ITS2 are shown by a thin line. Dots indicate gaps. High quality figures are available online.

ITS2 sequences of more than 120 *Anopheles* species deposited in GenBank were surveyed for the presence of repeat sequences by dot plot analyses. Consequently, *An. funestus* Giles (AF062512), *An. beklemishevi* Stegnii and Kabanova (AY593958), and *An. crucians* complex (species A, AY245553; species B, AY386963) were found to have repeats. *An. funestus* and *An. beklemishevi* had two repeats. *An. crucians* species B had three types of repeats with different sequence patterns. The number of repeats in each type was two, five, and three, respectively. The two repeats found in *An. crucians* species A had sequence similarities to the type found in *An. crucians* species B (Figure 5). These repeat sequences found in the ITS2 region of *Anopheles* species were 40–200 bp in length, and no sequence similarity was observed between species except for repeats found in the same species complex. Blast searches in GenBank did not reveal any DNA sequences having significant homology with the repeats. Apart from the repeat sequences in the *An. barbirostris* complex, secondary structures and inverted terminal sequences were not detected in the repeat sequences.

## Discussion

At 938 to 1729 bp, the ITS2 region of the five species in the *An. barbirostris* complex is extremely large. Sequence comparisons indicated that the unusual length of ITS2 was due primarily to sequences repeated tandemly. Considerable variation was observed in the number and formation of the repeat regions in the five sibling species of the *An. barbirostris* complex. The repeats could be classified into groups based on the observed variation in the sequences between species. With the exception of *An. barbirostris* A3, the other four species had repeats from the same group, implying that concerted evolution occurred between repeats of the ITS2 region in each species. ITS2 is part of the rDNA cistron, which is also repeated tandemly and undergoes concerted evolution (Brown et al. 1972; Arnheim et al. 1980; Tautz et al. 1987; Porter and Collins 1991). Thus, the repeat sequences in ITS2 are subjected to concerted evolution, at the level of being repeats themselves and as tandem repeats of rDNA. In concerted evolution, sequences of repetitive elements are homogenized, which has occurred in rDNA. However, the repeats in the ITS2 region of *An. barbirostris* could be classified into groups based on sequence differences. In addition, several repeat sequences belonging to different groups were arrayed in turn. For example, the ITS2 region of *An. barbirostris* species A4 consisted of four tandemly arrayed units consisting of a repeat from group 3 and one from group 8. It appears that concerted evolution occurs as a unit of two repeats in *An. barbirostris* species A2, A4, and *An. campestris*-like, and as one of three repeats in *An. barbirostris* species A1. Furthermore, some repeats of an ITS2 region do not appear subjected to concerted evolution. The three repeat sequences of *An.* *barbirostris* species A3 (i.e. A3-1, A3-2, and A3-3) were highly divergent, while A1-10, A2-8, and ca-8, all of which belong to different species, were related more closely than other groups of repeats in the same species. Molecular mechanisms, such as unequal crossing over, gene conversion, and transition have been proposed to explain the concerted evolution in rDNA (Smith 1976; Dover 1982, 2002; Eickbush and Eickbush 2007). Indeed, it is possible that different mechanisms operate on concerted evolution of repeats at these levels, i.e., at the level of the repeat itself and at the general level of the rDNA.

In the ITS2 region of *Anopheles* species, simple tandem repeats (2–5 bp in length) are observed frequently (Fritz et al. 1994; Cornel et al. 1996; Xu and Qu 1997; Jariyapan et al. 2005; Li and Wilkerson 2007). Variations in the type and number of simple repeats have been used to distinguish between species of the *An. dirus* complex (Xu and Qu 1997) and *An. albitarsis* complex (Li and Wilkerson 2007), as well as populations of *An. nuneztovari* Gabaldón (Fritz et al. 1994). These simple repeats are thought to arise partly due to slipped-strand mispairing (Levinson and Gutman 1987). In this study, it is shown that longer repeat sequences (> 30 bp) are included in the five sibling species of the *An. barbirostris* complex, and also in another four *Anopheles* species. These findings indicate that although the generation mechanism is unknown, the occurrence of repeats in ITS2 is not rare in *Anopheles* mosquitoes. For example, variation was observed in the number of repeats between the species A and B of *An. crucians* complex, and the other species of *An. crucians* complex were found to have no repeats in the ITS2 region (Wilkerson et al. 2004). Similarly, repeat sequences in the ITS2 region were found in *An. funestus* and *An. beklemishevi*, though several closely related species in the same group had no repeats in the region (Garros et al. 2004; Kampen 2005). While some long ITS2 sequences from *Anopheles* species have been deposited in the GenBank database, they do not contain repeat sequences. If the repeat sequences in the ITS2 region accumulated mutations, then repeat detection would prove difficult. It is thus possible that long ITS2 regions, that apparently lack repeat sequences, may have contained repeats previously, and the accumulated mutations resulted in some of these sequences becoming relics. Indeed, the three repeats in *An. barbirostris* species A3 are so divergent that finding any similarities between them is difficult without comparing them against the other four species in the *An. barbirostris* complex. The core secondary structure of ITS2 is conserved in a wide variety of eukaryotic taxa (Schultz et al. 2005). However, no such conservation of secondary structure has been observed in *Anopheles* mosquitoes to date. This may be one reason why the ITS2 region in some *Anopheles* species contains repeat sequences, which explains the observed size variation in related species.

Exclusion of the repeat sequences from the ITS2 region of the five species of *An. barbirostris* complex facilitated good alignment. The resulting phylogenetic trees showed that A3 was highly distinct from the other four sibling species, which implies that A3 may not be related to the other four species as proposed by Paredes-Esquivel et al. (2009). However, A3 had three repeat sequences that were homologous to the sequences found in the ITS2 region of another member of the *An. barbirostris* complex. In addition, the repeats in all five sibling species were inserted at the same position, implying that the ITS2 region of these species had a common origin. Furthermore, phylogenetic analysis of the mitochondrial COI and COII sequence data placed Al almost equally between A3 and the other species, indicating a lack of support for the separation of A3 from the other species obtained in the ITS2 region data. Although the phylogenetic status of A3 remains unclear, future analyses of sequence data in other species of the Barbirostris subgroup will further understanding of the taxonomic position of A3 in this species complex.

**Supplementary table.**
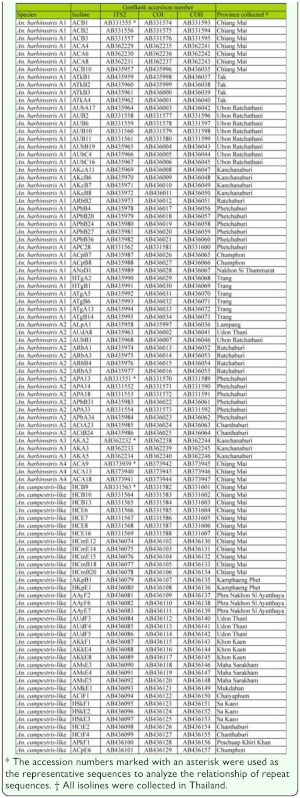
A list of isolines used in this study with their GenBank accession numbers and localities in Thailand.
